# A social justice lens on health inequalities: Implications and further considerations

**DOI:** 10.1016/j.puhip.2026.100734

**Published:** 2026-01-13

**Authors:** Kadek Suhardita, I Wayan Widana, Rikas Saputra, Erfan Ramadhani, Putu Ari Dharmayanti, Sri Datuti, Rr Dwi Umi Badriyah, Rizky Ananda Pohan, Ulfah, I Dewa Ayu Eka Purba Dharma, Laily Tiarani Soejanto, Arizona

**Affiliations:** a Universitas PGRI Mahadewa, Denpasar, Indonesia; b Universitas Islam Negeri Raden Fatah Palembang, South Sumatra, Indonesia; c Universitas PGRI Palembang, Palembang, Indonesia; d Universitas Pendidikan Ganesha, Indonesia; e Universitas Mahasaraswati Denpasar, Indonesia; f Institut Agama Islam Negeri Langsa, Langsa, Aceh, Indonesia; g Universitas Islam Nusantara Bandung, Indonesia; h Universitas PGRI Kanjuruhan Malang, Indonesia

**Keywords:** Socioeconomic health inequalities, Social justice, Health equity, Public health policy, Global health disparities, Adaptive interventions

Dear Editor,

In response to the article by Ref. [1] "Socio-economic health inequalities through the lens of social justice theory, an innovative perspective" offers a compelling analysis of how frameworks of social justice can shed light on the disparities in health outcomes among different populations. Its novel approach is especially pertinent in the context of today's globalized society, where socioeconomic factors increasingly influence access to healthcare and overall health. We commend the authors for their efforts in linking theoretical frameworks with empirical realities, thereby emphasizing structural inequalities that frequently remain overlooked [1]. Nonetheless, certain aspects, such as the relationship between local policies and global health trends, warrant a more thorough investigation. This correspondence seeks to broaden the discussion by considering wider implications, contextual adaptability, and possible pathways for applying theory in practice across various socio-political contexts.

At the heart of the article lies the claim that principles of social justice equity, fairness, and inclusivity should direct public health initiatives aimed at diminishing socioeconomic health disparities. This notion is becoming increasingly vital in a swiftly changing era characterized by technological advancements, urban growth, and demographic shifts [2]. Utilizing social justice theory offers a normative perspective to assess both the equity of resource allocation and the ethical responsibilities of policymakers. However, the applicability of findings is still constrained, as disparities appear differently across various contexts, cultures, and policy landscapes [3]. Tackling these issues necessitates adaptive approaches that are attuned to local circumstances while upholding a dedication to universal equity principles, highlighting the importance of flexible and context-aware public health strategies.

Recent studies have underscored both advancements and ongoing disparities in tackling socioeconomic health inequalities. Certain groups have reaped the benefits of focused interventions, enhanced healthcare access, and social support initiatives. Nevertheless, inequalities remain, especially within marginalized communities, frequently worsened by structural obstacles and systemic injustices [4]. The article implicitly recognizes these disparities, yet future research should investigate how platform design, healthcare systems, and social policies influence outcomes. Grasping these dynamics is essential for predicting unintended effects and optimizing beneficial outcomes. Furthermore, upcoming studies should consider cross-sector partnerships, merging education, housing, and labor policies to develop comprehensive solutions that target the fundamental causes of health inequities rather than merely addressing their symptoms [5].

A strategy for addressing socioeconomic health disparities entails the integration of multi-faceted approaches: reforming policies, engaging communities, and ensuring institutional accountability. Solutions should harmonize immediate interventions with enduring structural modifications, which encompass fair access to healthcare, education, and social protection systems [6]. The effective execution of these strategies necessitates human dedication, political determination, and participatory governance to guarantee that interventions are culturally relevant and sustainable. By highlighting the importance of collaboration among stakeholders from local communities to global organizations the article emphasizes the need for collective action [7]. In conclusion, converting social justice theory into actionable practice requires both structural changes and active human involvement, ensuring that ethical principles are woven into practical solutions aimed at diminishing health inequalities worldwide.

The consequences of this correspondence underscore the wider significance of incorporating social justice viewpoints into public health research and practice. This commentary highlights the originality of the initial article, especially its capacity to link theory with current health inequities. Its strengths encompass an innovative conceptual framework and an emphasis on systemic determinants, whereas its limitations pertain to contextual generalizability and empirical validation across various populations [8]. Future initiatives should focus on cross-cultural studies, longitudinal analyses, and policy evaluations to enhance the applicability of social justice frameworks [9]. By connecting theory with practice, both the article and this commentary jointly advance a more equitable approach to health policy, fostering ongoing discussions on practical solutions for mitigating socioeconomic health disparities globally.

## Funding

The authors declare that no funding was received for this paper.

## Declaration of competing interest

The authors declare that they have no known competing financial interests or personal relationships that could have influenced the work reported in this paper.
